# Rural opioid stewardship: Lessons learned from one community and the retirement of a pain management clinician

**DOI:** 10.1016/j.pmedr.2025.103124

**Published:** 2025-05-31

**Authors:** Holly Ann Russell, Jade Malcho, Michele Lawrence

**Affiliations:** a Department of Family Medicine and Center for Community Health and Prevention, University of Rochester School of Medicine and Dentistry, Rochester, NY 14642, USA; b Department of Psychiatry & Emergency Medicine, University of Rochester School of Medicine and Dentistry, Rochester, NY 14642, USA; c Department of Psychiatry, University of Rochester School of Medicine and Dentistry, Rochester, NY 14642, USA

**Keywords:** Pain, Substance, Rural, Opioid, Drug

## Abstract

**Objective:**

This commentary offers a perspective of a rural community's response to a dissolution of a pain management practice with an emphasis on how to avoid serious harm for patients on opioid therapy when continuity of chronic pain management care is interrupted.

**Methods:**

The ‘Ecosystem of Recovery”9 framework was utilized to explore the response of rural communities to pain management clinic closures that includes community engagement, incorporation of a multi-disciplinary team, clinician education and consultative support to deliver evidence-based care while triaging patient needs. This project was undertaken as a quality improvement initiative and did not meet the definition of research according to 45CFR46, the federal law dictating human subjects research in the US.

**Results:**

Community engagement resulted in a unified, multi-disciplinary team-based approach that supported existing medical infrastructure to transition patients seeking continuation of care. Emergency Departments linked patients to existing primary care offices, behavioral health and addiction medicine specialists based on patient presentation. Education and consultation efforts supported triage of their acute medical needs and linkage to appropriate level of care. Buprenorphine initiation to treat opioid withdrawal and harm reduction strategies for overdose prevention, including naloxone, were included at the core of the strategies.

**Conclusions:**

Implementation of clinician and patient support strategies are recommended when gaps in pain management treatment occur in resource-limited communities. We share our coordinated community-based approach for patients who are navigating this care transition, while educating and supporting clinicians how to evaluate and initiate treatment for opioid withdrawal and opioid use disorder.

## 1 Setting the stage

In November 2021, a pain management clinician with 600 patients in rural upstate New York announced their retirement. Most of their patients had complex chronic pain and were prescribed a combination of controlled substances to help manage their symptoms. The community struggled to link the patients to ongoing care.

In this commentary, we explore how the region responded as well as insights and opportunities for improvement at both a state and federal level.

## 2 Introduction

Chronic pain is estimated to impact 18–20 % of the United States (U.S.) adult population and 30.9 % of rural residents (Yong et al., 2022). Risk factors for chronic pain are more prevalent in rural communities and rural residents are more likely to be prescribed opioid medication than their non-rural counterparts (Pitcher et al., 2019; Golembiewski et al., 2022; Rafferty et al., 2021; Parchman et al., 2020). These risk factors include high levels of poverty and higher rates of labor-intensive work putting those at higher risk for accidents, musculoskeletal injuries and developing chronic pain syndromes (Pitcher et al., 2019; Golembiewski et al., 2022; Rafferty et al., 2021).

Numerous barriers exist to accessing health care services in rural communities including commuting through a challenging physical environment, economic limitations, and stigma related to substance use disorder (SUD)treatment (Golembiewski et al., 2022; Rafferty et al., 2021). There also is a shortage of primary care providers (PCPs) and specialists which creates accessibility limitations to general and specialty care (Parchman et al., 2020).

In the 2022 updated guidelines from the Centers for Disease Control and Prevention, challenges accessing multidisciplinary pain programs are noted. “The range of therapeutic options has historically been inaccessible to many patients because of factors such as inadequate clinician education, training, and guidance; unconscious bias; a shortage of pain management specialists; insufficient access to treatment modalities such as behavioral therapy; siloed health systems; insurance coverage and reimbursement policies; and lack of clarity about the evidence supporting different pain treatments.” (Dowell, 2022)

The current U.S. healthcare system disincentivizes caring for the most complex and vulnerable populations through a fee-for-service reimbursement plan that highly prioritizes procedures and quality metrics. This construct does not consider the effects of underlying social determinants of health and does not support trauma-informed management-based care that balances pharmacologic and non-pharmacologic therapies (Sinha and Dineen Gillespie, 2022; Fiscella and Epstein, 2008).

Despite it being established that long-term opioid therapy should not be discontinued or rapidly reduced abruptly, it still occurs (Agnoli et al., 2021). Continuity of treatment for patients on chronic opioid therapy must be considered to avoid negative outcomes including opioid withdrawal, SUD, increased pain, suicide and opioid overdose deaths which are associated with abrupt tapering of opioids (Agnoli et al., 2021).

While there is strong evidence to support the integration of behavioral health, including SUD treatment, within primary care, this is not generally achieved due to staffing shortages and non-existent funding mechanisms (Balasubramanian et al., 2017). Additionally, legal and regulatory changes with good intentions of addressing the opioid overdose epidemic, have unintentionally incentivized clinicians to avoid prescribing opioids who are already on chronic opioids from other prescribers (Dowell, 2022).

## 3 Our approach

Recognizing that a gap in rural access to treatment was imminent in December 2021, our first step to identify a central champion who could bring all the appropriate parties to the table as the crisis evolved. Our champion was a Senior Vice-President level health care administrator with years of experience working in the community. Immediate solutions were based on an existing institutional program called the “Ecosystem of Recovery” (Lawrence, 2025) and included the following goals:

1. Engagement of the Community: It was fundamental to engage the local rural PCPs and specialists across the regions and to ensure proper community support. With their needs in mind, in February of 2022 we initiated discussions with the New York State (NYS) Department of Health and federal government leaders to explore whether there was a state or nation-wide pain management team that could be activated. We recognized that most patients would need to have an evaluation by a pain management specialist, which could not be done by a NYS SUD treatment center due to license restrictions.
2. Development of a Local Multi-Disciplinary Committee: As there was not a currently active federal or state response team, throughout the late winter and early spring of 2022, team members were recruited across the region including some of the remaining pain management specialists, psychiatrists and psychiatric nurse practitioners, addiction medicine specialists, and primary care offices including nursing and administrative staff to make up the Pain Management Advisory Committee. This group identified available capacity to accept patients and promoted education for clinicians to continue pain management medications as well as to initiate buprenorphine for opioid use disorder. The “Ecosystem of Recovery” program assisted with training Behavioral Health Assessment Officers (BHAOs) and placed two BHAO's in local rural emergency departments as a supplemental workforce to engage patients with behavioral health concerns. The BHAO program was expanded to evaluate patients with potential SUD and coordinate initiating buprenorphine in the emergency department and linkage to treatment.
3. Triage of Patients Treatment Needs: The Pain Management Advisory Committee helped to triage patients based on the efficacy of their pain treatment and presence or absence of SUD. Starting in the Spring of 2022 the committee met biweekly for about six weeks until the situation stabilized and then met monthly for the remainder of the year with medical directors from the two large regional Federally Qualified Health Centers to link patients to primary care or SUD treatment as appropriate.
4. Emphasis on Harm Reduction Strategies**:** In some rural communities, emergency medical services and police may not carry naloxone and/or may be more restrictive on its distribution. In our case, we found that zero out of three rural community hospitals were registered to provide free naloxone to patients through state funding. We aimed to prepare rural emergency departments to distribute naloxone to any patient with opioid use and treat any patient in withdrawal with buprenorphine.
5. Implementation of Innovative Education & Consultation Models: In May of 2022, we invited the rural PCPs to attend an out-of-state weekly addiction medicine case conference. Unfortunately, with the lack of familiarity of NYS regulations, not all recommendations could be implemented, and the timing was challenging for the PCPs to routinely attend. In January 2023 a rural pilot project was implemented to facilitate review of patient cases by a consulting team that included a pharmacist, pain management physician, and addiction medicine physician from the region. To date, 16 cases have been reviewed, many patients whose care was transferred to a PCP from the pain clinic.

## 4 Discussion

Closures of pain management clinics and retirements of experienced clinicians are inevitable and can exacerbate the existing disparities in access faced by rural communities. While some patients may have come to develop an underlying SUD, they did not write their opioid prescriptions themselves, and it is the system's responsibility to support them in diagnosis and long-term treatment in the new absence of their pain management prescriber.

Early in our approach, it was recognized that state and federal resources did not currently exist, and the local Pain Management Advisory Committee was created to provide support to the local offices triaging complex patient needs presenting to them. Challenges included engaging all potentially receiving sites and efforts to track or proactively manage specific patient transitions were also limited as the pain management practice verbally provided information on the number of impacted patients, we were not granted access to patient level detail.

We successfully introduced processes to support patients in three rural emergency department which included initiation of medication for opioid use disorder, naloxone delivery and BHAO assessments for patients who find themselves seeking care in these safety net settings.

Education and clinical decision support delivered via consultative specialty teams case reviews only bridged some of the gaps in standard of care as even the best educated and resourced PCPs are generally unable to accept large numbers of patients onto their panel urgently since most rural practices operate at or over capacity.

Recommendations for future management of similar scenarios were later noted in the 2022 NYS Settlement Fund Advisory Board annual report, “Funding will be appropriated to the Department of Health to create a rapid response Telehealth and outreach program to be on the ready when a closure happens to get patients adequate resources to avoid overdoses and other bad health outcomes.” (*Opioid Settlement Fund Advisory Board Annual Report*, 2022)

## 5 Conclusions

Having strategies in place to support communities when access to medical treatment is disrupted is important to decrease potential harm that patients face when situations such as these occur. Future considerations include the creation of a national pain management response team that can be activated quickly by designated agencies and remain on the ground for several months while a local response is coordinated. Additionally, improving the capacity of PCPs that require reimbursement policy and guideline revisions that more adequately support the delivery of comprehensive pain management services in primary care settings are needed to safely manage these prescriptions and to care for patients with complex chronic pain. There should be a focus on increasing competence for leaders identified in key specialties across the healthcare delivery system including pain management, emergency medicine, primary care and addiction medicine who are knowledgeable about both chronic pain and addiction who can be ready to educate their own teams and be prepared to respond to these events.

## CRediT authorship contribution statement

**Holly Ann Russell:** Writing – original draft, Conceptualization. **Jade Malcho:** Conceptualization, Writing – review & editing. **Michele Lawrence:** Writing – original draft, Project administration, Conceptualization.

## Authorship

Holly Ann Russell, MD, MS; Jade Malcho, MD; and Michele Lawrence, MBA, MPH have made substantial contributions to all of the following:

1. The conception and design of the study, or acquisition of data, or analysis and interpretation of data.
2. Drafting the article or revising it critically for important intellectual content.
3. Final approval of the version to be submitted.

## Funding

This article was funded by the U.S. Department of Health and Human Services, Health Resources and Services Administration under cooperative agreement number UD9RH33632. The views expressed in this publication are solely the opinions of the authors and do not necessarily reflect the official policies of the U.S. Department of Health and Human Services, or the Health Resources and Services Administration nor does mention of the department or agency names imply endorsement by the U.S. Government.

## Declaration of competing interest

The authors declare that they have no known competing financial interests or personal relationships that could have appeared to influence the work reported in this paper.

## Data Availability

No data was used for the research described in the article.
